# The relationship between pain with walking and self-rated health 12 months following total knee arthroplasty: a longitudinal study

**DOI:** 10.1186/s12891-017-1430-7

**Published:** 2017-02-10

**Authors:** Maren Falch Lindberg, Tone Rustøen, Christine Miaskowski, Leiv Arne Rosseland, Anners Lerdal

**Affiliations:** 1 Department of Surgery, Lovisenberg Diakonale Hospital, Pb 4970 Nydalen, 0440 Oslo, Norway; 2 Department of Nursing Science, Institute of Health and Society, Faculty of Medicine, University of Oslo, Pb 1072 Blindern, 0316 Oslo, Norway; 3 Department of Research and Development, Division of Emergencies and Critical Care, Oslo University Hospital, Pb 4956 Nydalen, 0424 Oslo, Norway; 4 School of Nursing, University of California, San Francisco, UCSF, Box 0610, San Francisco, CA 94143 USA; 5 Institute of Clinical Medicine, University of Oslo, Pb 1072 Blindern, 0316 Oslo, Norway

**Keywords:** Self-rated health, Walking, Postoperative pain, Total knee arthroplasty, Fatigue

## Abstract

**Background:**

A subgroup of patients continue to report pain with walking 12 months after total knee arthroplasty (TKA). The association between walking pain and self-rated health (SRH) after TKA is not known. This prospective longitudinal study aimed to investigate the association between a comprehensive list of preoperative factors, postoperative pain with walking, and SRH 12 months after TKA.

**Methods:**

Patients (*N* = 156) scheduled for TKA completed questionnaires that evaluated demographic and clinical characteristics, symptoms, psychological factors, and SRH. SRH was re-assessed 12 months after TKA. Clinical variables were retrieved from medical records. Pain with walking was assessed before surgery, at 6 weeks, 3, and 12 months after TKA. Subgroups with distinct trajectories of pain with walking over time were identified using growth mixture modeling. Multiple linear regression was used to investigate the relationships between pain with walking and other factors on SRH.

**Results:**

Higher body mass index, a higher number of painful sites at 12 months, recurrent pain with walking group membership, ketamine use, higher depression scores, and poorer preoperative self-rated health were associated with poorer SRH 12 months after TKA. The final model was statistically significant (*p* = 0.005) and explained 56.1% of the variance in SRH 12 months after surgery. SRH improved significantly over time. Higher C-reactive protein levels, higher number of painful sites before surgery, higher fatigue severity, and more illness concern was associated with poorer preoperative SRH.

**Conclusions:**

In patients whose walking ability decreases over time, clinicians need to assess for unreleaved pain and decreases in SRH. Additional research is needed on interventions to improve walking ability and SRH.

## Background

Osteoarthritis (OA) of the knee is a common disorder that leads to pain and restricted mobility. Total knee arthroplasty (TKA) for OA aims to relieve pain and improve function when conservative treatment is no longer effective. Although the procedure is highly effective in terms of prosthesis-related outcomes [1] and quality of life [2], some patients continue to report a higher degree of functional limitations 1 year after TKA compared to healthy age matched controls [3]. Of note, in a recent systematic review [4], approximately 20% of patients reported little or no improvement in terms of pain relief.

The mechanisms that underlie this variability in TKA outcomes are likely to be multifactorial and complex [5]. For example, poorer self-efficacy with walking skills [6], more preoperative walking limitations, higher body mass index (BMI), slower 1 month gait speed, contralateral knee pain, and use of quadstick before surgery [7] were associated with poorer walking outcomes 6 months after TKA. Recently, our research group demonstrated that one in five patients reported no improvements in pain interference with walking 1 year after TKA [8]. In that study, pain interference with walking was measured at repeated intervals during the first year. In a growth mixture modeling, two subgroups of patients with different trajectories of pain with walking were identified. While one group of patients (i.e., Continuous Improvement group, 78%) improved steadily during the first year, a second group of patients (i.e., Recurrent Interference group, 22%) was characterized by initial improvements followed by recurrent pain interference with walking after the first 3 months, resulting in no improvements in terms of pain interference with walking 12 months after surgery. Patients in the Recurrent Interference group were characterized by higher preoperative pain, fatigue, and depression scores and poorer illness perceptions compared to Continuous Improvers. The current study builds on these results to further investigate how walking outcomes and a comprehensive set of predictors are related to a general health outcome (i.e., self-rated health (SRH) at 12 months after TKA.

Walking difficulties seem to influence patients’ future health. For example, walking difficulties were associated with higher mortality rates 10 years after TKA [9]. In addition, walking difficulty and shorter walking distance predicted higher health care costs, disability, and mortality [10] as well as poorer self-rated health [11] in the general population.

SRH is a global measure of general health status [12] and is considered a reliable predictor of a variety of outcomes including mortality and morbidity, recovery after disease, and use of health care resources in various patient populations [13–15]. In addition, SRH is used as an outcome measure in population-based studies [16] and was associated with walking speed and walking difficulties [11]. Because SRH was responsive to changes in health and mental well-being during recovery after TKA [17], Peruccio and colleagues suggested that the use of this simple measure could identify patients who might benefit from targeted interventions to improve general health. However, to our knowledge, no studies have investigated the association between pain with walking, other pre- and perioperative factors, and SRH 1 year after TKA. Therefore, the aims of this study were to (1) describe changes in SRH from before until 12 months after TKA, (2) identify preoperative factors associated with poorer preoperative SRH, and (3) investigate the key predictors of poorer SRH 12 months after TKA.

## Methods

Data collection for this longitudinal study was performed between October 2012 and September 2014 at a high volume surgical clinic in Norway. The clinic serves patients from all regions of Norway. Patients ≥18 years of age were eligible for inclusion if they were scheduled for TKA for OA, agreed to participate, and did not have a diagnosis of dementia. Patients undergoing unicompartmental or revision surgery were excluded. The study was approved by the Regional Medical Research Ethics Committee of Health South East of Norway (# 2011/1755).

### Patients and procedures

Written information was distributed to all patients considered for eligibility prior to or on the day of admission. A nurse approached patients who met the inclusion criteria and invited them to participate. Written consent regarding questionnaires and extraction of data from the medical records was obtained from all patients before completing the baseline questionnaire that assessed demographic, clinical, symptom and psychological characteristics, as well as health-related quality of life and pain interference with walking. Follow-up assessments of pain interference with walking were performed at postoperative day (POD) 4, 6 weeks, 3 months, and 12 months after surgery. Health-related quality of life follow-up questionnaires were completed 12 months after surgery. All questionnaires were returned in sealed envelopes. Non-responders received a reminder by mail or phone.

### Perioperative procedures

The surgical department had a standardized regimen for anesthesia, surgery and postoperative pain management. All patients received the same posterior cruciate-retaining fixed modular-bearing implant for the TKA using an intraoperative tourniquet and with drains that were removed on POD 1. The full regimen for pain management was described in detail elsewhere [8]. Patients were allowed full weight bearing starting on POD 1 and were followed up with weekly physical therapy for 4 to 6 months after surgery. Most patients were prescribed a combination of acetaminophen and tramadol at discharge.

### Measurements

Self-rated health: Patients rated their own perceived overall health status today (i.e., SRH) on a numeric rating scale from the well validated EuroQol-5D-3 L [18]. The scale ranges from 0 (worst imaginable health status) to 100 (best imaginable health status).

### Clinical variables

Data on comorbidities, BMI, American Society of Anesthesiologists (ASA) classification score, blood pressure, type of anesthesia, type of implant, length of surgery, length of stay, C-reactive protein (CRP), and creatinine values were extracted from the medical records. Data on opioid consumption and use of ketamine were obtained through chart review.

### Pain and interference with function measures

*The Brief Pain Inventory* (BPI) was used to measure pain and interference with walking [11]. The BPI consists of four items that evaluate pain intensity rated on a numeric rating scale (NRS) from 0 (no pain) to 10 (pain as bad as you can imagine); seven items that evaluate pain interference with seven domains of life (i.e., general activity, mood, walking ability, normal work, relations to other people, sleep, enjoyment of life) rated from 0 (no interference) to 10 (interferes completely); and a body map that evaluates pain locations. The validity and reliability of the Norwegian version of the BPI is well established [12].

For this study, pain interference with walking was a factor of particular interest and was assessed on the day before surgery, on POD 4, at 6 weeks, 3 months, and 12 months after surgery. This final endpoint was chosen because the largest gains in functional improvement typically occur within 1–3 years after TKA [19, 20]. In our previous study [21], two subgroups of patients with distinct trajectories of pain interference with walking following TKA were identified. The first group (i.e., Continuous Improvement group) reported significant improvements in pain with walking from before to 12 months after surgery. The second group (i.e., Recurrent Interference group) reported significant improvements in pain interference with walking from before until 3 months after surgery, followed by a distinct worsening returning to preoperative scores 12 months after surgery. This subgroup classification was included in this study as a potential predictor of SRH. Details about this subgroup analysis are published elswhere [21].

### Symptom measures

#### Fatigue severity

Fatigue severity was evaluated using the 5-item Lee Fatigue Scale (LFS). Each item was rated on a 0 to 10 NRS. A mean score using all items was calculated with higher scores indicating higher fatigue severity. The LFS has satisfactory validity and reliability [13]. In this study, its Cronbach’s alpha was .92.

#### Fatigue interference

Fatigue interference during the past week was measured using the 7-item Fatigue Severity Scale (FSS-7). Patients used a 7 point Likert scale to rate their agreement with 7 statements. A total mean score using all seven items was calculated. Scores can range from 1 to 7 with higher scores indicating higher levels of interference. Psychometric properties of the Norwegian version of the FSS-7 were good [14]. In this study, its Cronbach’s alpha was .92.

#### Mood states

The Hospital Anxiety and Depression Scale (HADS) [15] was used to evaluate symptoms of depression and anxiety. HADS consists of two subscales with 7 items for depression and 7 items for anxiety. Separate mean scores for anxiety and depression were calculated, using all the specific items for each subscale [22]. Each subscale can range from 0 to 21 with higher scores indicating higher levels of anxiety and depression. The Norwegian version of the HADS has excellent psychometric properties [16]. In this study, the Cronbach’s alphas for the depression and the anxiety subscales were .78 and .86, respectively.

### Psychological measure

*The Brief Illness Perception Questionnaire* (BIPQ) [17] was used to measure self-reported perceptions of illness. The scale consists of eight items that each measure one dimension of illness perceptions (i.e., consequences, timeline, personal control, treatment control, identity, illness concern, coherence, and emotional response). Each item was rated on a 0 to 10 NRS. For this study, the patients rated their illness perceptions in relation to their OA knee. Five items from the BIPQ (i.e., consequences, personal control, identity, concern, emotional response) were used in the statistical analyses because these specific items were sensitive to changes over time in patients with traumatic injuries [18].

### Data analysis

Data analysis was performed using Statistical Package for Social Science version 22 (IBM, Armonk, NY). Growth mixture modeling (GMM) with full maximum likelihood estimation using Mplus Version 7.3 [23] was performed to identify subgroups of patients with distinct trajectories based on their experience with pain interference with walking before through 12 months after TKA. The statistical procedure for this analysis is described in detail elsewhere [21].

For the current analysis, descriptive statistics were generated on sample characteristics, pain, and health related quality of life. A paired t-test was done to evaluate for changes in SRH from prior to until 12 months after TKA. Exploratory analyses using Pearson’s bivariate correlation coefficients were performed to select potential variables for inclusion in the multivariate analyses. Non-significant variables were excluded from further testing. Separate regression analyses were performed for preoperative SRH and SRH at 12 months. For both analyses, age, sex and number of comorbidities were included as covariates since these variables were associated with SRH in various populations [24–26].

#### Preoperative SRH

Variables with a statistically significant association with preoperative SRH in the exploratory analysis (Table 1) were entered into a blockwise multiple linear regression using backwards entry in the following blocks: demographic characteristics, clinical characteristics, pain characteristics, preoperative symptoms, and psychological characteristics. Then, variables with the least association (i.e. highest *p*-value) with the dependent variable were excluded one by one and nested models were repeated until the final model was determined.

**Table 1:** Potential predictors to self-rated health prior to surgery and 12 months following total knee arthroplasty – Pearsons bivariate correlation coefficient

	Pearsons correlation coefficient			
	Prior to surgery	P-value	12 months	P-value
Demographic characteristics				
Age	0.21^+^	**	0.19^+^	*
Sex (male as reference)	−0.20^+^	*	0.02^+^	
Cohabitation status (living with partner as reference)	0.08		−0.03	
Employment status (paid work as reference)	0.11		0.01	
Education level (less than 14 years as reference)	0.01		0.04	
Clinical characteristics				
Body mass index	−0.18^+^	*	−0.30^+^	**
Number of comorbidities (0–5)	−0.20^+^	*	−0.26^+^	**
American Society of Anesthesiologists’ physical status classification (1–3)	0.01		−0.05	
Systolic blood pressure	0.17^+^	*	0.09	
C-reactive protein	−0.30^+^	**	−0.15	
Creatinine	0.17^+^	*	0.08	
Pain characteristics				
Average pain prior to surgery	−0.31^+^	**	−0.16^+^	*
Worst pain prior to surgery	−0.35^+^	**	−0.31^+^	**
Pain interference with function prior to surgery	−0.43^+^	**	−0.36^+^	**
Number of painful sites prior to surgery	−0.33^+^	**	−0.27^+^	**
Number of painful sites 12 months	n/a		−0.35^+^	**
Contralateral knee pain 12 months	n/a		−0.06	
Pain interference with walking group (Continuous improvement group as reference)	n/a		−0.55^+^	**
Perioperative characteristics:				
Type of anesthesia (regional anesthesia as reference)	0.01		−0.17^+^	*
Length of surgery (minutes)	n/a		−0.06	
Length of stay (days)	n/a		−0.06	
Ketamine (no as reference)	n/a		−0.24^+^	**
Average dose of opioids over 4 days	n/a		−0.10	
Preoperative symptoms				
Fatigue severity (Lee fatigue scale total score)	−0.44^+^	**	−0.33^+^	**
Fatigue interference (Fatigue Severity Scale)	−0.38^+^	**	−0.36^+^	**
Depression	−0.32^+^	**	−0.43^+^	**
Anxiety	−0.32^+^	**	−0.31^+^	**
Psychological characteristics***				
Consequences	−0.35^+^	**	−0.21^+^	**
Personal control	−0.20^+^	*	−0.31^+^	**
Identity	−0.29^+^	**	−0.21^+^	**
Illness concern	−0.43^+^	**	−0.31^+^	**
Emotional response	−0.37^+^	**	−0.23^+^	**
Health characteristics				
Self-rated health prior to surgery	n/a		0.46^+^	**

*Correlation is significant at the 0.05 level (2 tailed)

** Correlation is significant at the 0.01 level (2 tailed)

*** Single item scores from the Brief Illness Perception Questionnaire

n/a = not applicable

^+^Selected for inclusion in linear regressions

#### SRH 12 months

Variables with a statistically significant association with SRH at 12 months in the exploratory analysis (Table 1) were entered into a blockwise multiple linear regression using backwards entry using the following blocks: demographic characteristics, clinical characteristics, pain characteristics, perioperative characteristics, preoperative symptoms, psychological characteristics, and health characteristics. Then, variables with the least association (i.e. highest *p*-value) with the dependent variable were excluded one by one and nested models were repeated until the final model was determined. For both analyses, the blocks were created based on the framework used in our previous studies [21, 27, 28] that investigated the impact of demographic, clinical, symptom, and psychological characteristics on acute pain and pain interference with walking after TKA.

## Results

### Demographic and clinical characteristics

A total of 245 patients were invited to participate in the study, of which 33 declined to participate and six had their surgery cancelled. A total of 206 consenting patients were enrolled in the study. Two patients were later excluded due to postoperative disorientation, one patient died, and 47 had incomplete data on several of the variables of interest, leaving a total of 156 patients in this analysis. No significant differences were found between responders and non-responders based on any of the preoperative characteristic used in this study.

As displayed in Table 2, the majority of the sample was female (67.9%), lived with a partner (61%), and were not employed (64%). The mean age was 69 (SD 9.1) years and 49% had completed higher education. SRH improved significantly over time (*p* < .001, *t* = 9.15) from before surgery (mean 60.9, SD 19.7) until 12 months after surgery (mean 74.7, SD 16.0).

**Table 2:** Demographic, clinical, symptom, and psychological characteristics of patients (*N* =156) prior to surgery

Demographic characteristics		Mean	SD
Age	Years	68.9	9.1
		n	%
Sex	Female	106	67.9
Cohabitation status	Married/partnered	95	60.9
Employment status	Unemployed/retired	100	64.1
Education level	College/university	76	48.7
Clinical characteristics		Mean	SD
	Body mass index (BMI)	29.2	4.6
		n	%
	Obese (BMI ≥30 kg/m^2^)	61	39.1
	Morbidly obese (BMI ≥40 kg/m^2^)	4	2.6
		Mean	SD
	Number of comorbidities (0–5)	1.2	1.0
	American Society of Anesthesiologists’ physical status classification score	2.0	0.5
	Systolic blood pressure	138.6	16.1
	C-reactive protein	3.3	3.0
	Creatinine	77.6	23.0
Pain characteristics			
	Average pain prior to surgery (0–10)	5.3	1.8
	Worst pain prior to surgery (0–10)	5.5	2.1
	Pain interference with function (0–10)	4.4	2.0
Number of painful sites	Prior to surgery	2.1	1.6
	12 months after surgery	2.0	1.2
		n	%
	Contralateral knee pain 12 months – yes (*n* = 130)	40	30.8
Pain interference walking group			
	Recurrent interference group	36	23.1
	Continuous improvement group	120	76.9
Perioperative characteristics:			
Anesthesia	Regional anesthesia	132	84.6
	Total intravenous anesthesia	24	15.4
		Mean	SD
	Length of surgery (minutes)	65	13.0
	Length of stay (days)	4.6	1.1
Pain management	Average dose of opioids (mg)^a^	12.6	6.2
		n	%
	Ketamine - yes	22	14.1
Preoperative symptoms		Mean	SD
	Fatigue severity (1–10)	2.6	2.2
	Fatigue interference (1–7)	3.9	1.5
	Depression (0–21)	3.4	3.1
	Anxiety (0–21)	4.6	3.7
Psychological characteristics^b^			
	Consequences (0–10)	6.2	1.8
	Personal control (0–10)	5.4	2.3
	Identity (0–10)	6.6	1.7
	Concern (0–10)	5.1	2.6
	Emotional response (0–10)	4.5	2.6
Health status			
	Preoperative self-rated health	60.9	19.7
	Self-rated health, 12 months	74.7	16.0

^a^All opioids were converted to intravenous morphine equivalents. Value is the average dose of opioids over 4 days

^*b*^Single item scores from the Brief Illness Perception Questionnaire

### Predictors of SRH prior to surgery

The final model for preoperative SRH is shown in Table 3. The final model was statistically significant (*p* = .002) and explained 37.0% of the variance in SRH prior to surgery. Higher levels of CRP, a higher number of painful sites before surgery, higher fatigue severity, and higher illness concern were significantly associated with poorer SRH, after controlling for age, sex, and number of comorbidities.

**Table 3:** Multiple linear regression analyses of demographic, clinical, symptom, and psychological factors on pre- and postoperative self-rated health in patients undergoing total knee arthroplasty (TKA)

Independent variables	*Beta*	B	*95% CI*	Explained variance (*R* ^2^)	Change of variance (*R* ^2^ –change)	*P-value*
Self-rated health prior to surgery						
*Step 1. Demographic characteristics*				8.5%	8.5%	.001
Age	.06	.12	−.18 to .42			
Sex (male as reference)	−.08	−3.39	−9.16 to 2.39			
*Step 2. Clinical characteristics*				18.2%	9.7%	<.001
Number of comorbidities	−.05	−1.02	−3.69 to 1.66			
C-reactive protein	**−.25**	**−1.63**	−2.51 to−.74			
*Step 3. Pain characteristics*				25%	6.8%	<.001
Number of painful sites before surgery	**−.18**	**−2.17**	−3.88 to−.46			
*Step 4 Preoperative symptoms*				32.7%	7.7%	<.001
Fatigue severity (higher = more fatigue)	**−.22**	**−1.99**	−3.45 to−.54			
*Step5 Psychological characteristics*				37.0%	4.3%	.002
Illness concern (higher = more concern)	**−.24**	**−1.82**	−2.97 to−.66			
Self-rated health 12 months after TKA						
*Step 1. Demographic characteristics*				3.5%	3.5%	.12
Age	−.02	−.04	−.27 to .20			
Sex (male as reference)	.06	1.99	−2.53 to 6.52			
*Step 2. Clinical characteristics*				15.8%	12.3%	<.001
Body mass index	**−.15**	**−.53**	−.98 to−.07			
Number of comorbidities	.07	−1.11	−3.19 to .96			
*Step 3. Pain characteristics*				42.9%	27.1%	<.001
Number of painful sites 12 months after surgery	**−.17**	**−2.18**	−3.93 to−.42			
Pain interference walking group (Continuous improvement group as reference)	**−.33**	**−12.45**	-.17.67 to -7.24			
*Step 4 Perioperative characteristics*				45.3%	2.4%	.025
Ketamine Yes/No – No as reference	**−0.15**	**−6.95**	−12.80 to −1.10			
*Step 5 Preoperative symptoms*				51.0%	5.7%	<.001
Depression	**−.21**	**−1.08**	−1.80 to **−**.37			
*Step 6 Psychological characteristics*				52.9%	1.8%	.04
Personal control	**−**.12	**−**.84	−1.74 to .07			
*Step 7 Health characteristics*				56.1%	3.2%	.005
Preoperative self-rated health	**.21**	**.17**	.05 to .29			

Statistically significant defined as *p* ≤ 0.05. Bold coefficient = statistically significant

*Abbreviations*: *Beta* standardized beta coefficient, *B* unstandardized coefficient, *CI* confidence interval, *SE* standard error

### Predictors of SRH at 12 months after TKA

The final model for SRH 12 months after surgery is shown in Table 3. The final model was significant (*p* = 0.005) and explained 56.1% of the variance in SRH 12 months after surgery. Higher BMI, higher number of painful sites 12 months after surgery, Recurrent Interference group membership, perioperative use of ketamine, higher preoperative depression score, higher perceived personal control, and poorer preoperative SRH were associated with poorer SRH 12 months after TKA, after controlling for age, sex and number of comorbidities. Among all associated factors, pain interference walking group membership had the strongest association with SRH at 12 months (*Beta =* −0.33). Patients who had no improvement in pain with walking scored 12.45 points (CI: −17.67 to −7.24) lower on SRH 12 months after TKA, compared to patients whose pain with walking decreased.

## Discussion

This study aimed to investigate key predictors associated with poorer SRH 12 months after TKA. Findings from this study suggest that lack of improvement in walking during the first year following TKA was the strongest predictor of poorer SRH scores 12 months after surgery. After controlling for demographic, clinical, and personal factors, membership in the Recurrent Interference group was associated with a 12 point lower SRH score, well above the minimally clinically important difference of 8 points for this scale [29]. In addition, a number of pre- and perioperative factors (i.e., higher number of painful sites, higher BMI, perioperative use of ketamine as supplemental pain medication, higher preoperative depression scores and poorer preoperative SRH) were associated with poorer SRH scores after TKA. In contrast, poorer preoperative SRH was associated with higher levels of CRP, a higher number of painful sites, higher fatigue severity, and more illness concern related to the osteoarthritic knee.

The preoperative SRH scores in our sample were comparable to OA patients awaiting joint replacement and TKA patients [30, 31]. As expected, overall the sample’s SRH score improved significantly during the first year, with a large effect size (Cohen’s d = 0.77). An improvement of almost 14 points is well above the clinically important difference of 8 points for this scale [29], which suggests that this improvement is clinically meaningful. This result is in line with previous studies that suggested that TKA has a high success rate for the majority of patients [2]. The improvement in SRH found at 1 year after surgery is similar to a previous report [31].

Consistent with other studies [9, 32], the association between poorer trajectories of pain interference with walking and poorer SRH 1 year after TKA suggests that walking ability is an important predictor of future health. For example, postoperative walking disability was linked to higher mortality rates 10 years after TKA [9]. In the general population, poorer walking performance among older adults was associated with a higher risk for future incidence of cardiovascular disease, mortality, and mobility disabilities [32]. Patients in our study received physical therapy for up to 6 months after surgery. While rehabilitation programs for TKA patients tend to focus on muscle strengthening and range of motion of the joint, assuming that this approach will in turn improve patients’ ability to walk, these programs seem to have limited effect on walking ability [33]. Although physical activity levels tend to increase after TKA, patients do not reach the same functional levels as the healthy population, and postsurgical activity levels seem to be influenced mainly by patients’ physical activity levels prior to surgery [34]. However, a recent study of a rehabilitation program that used walking and weight bearing exercises had better results for walking speed six months after TKA compared to usual physical therapy [35]. In addition, a 3 month hiking program resulted in improved walking speed and quality of life [36]. Improvements in walking and pain are among the most important outcomes for patients [37], but a substantial number of patients experience reduced walking distance and difficulty climbing stairs [38]. These persistent problems may potentially lead to a less active life and reduced quality of life. Additional studies are needed to determine the effect of improved walking skills on SRH after TKA.

Interestingly, higher preoperative BMI was associated with poor SRH at 12 months after surgery, but not with SRH preoperatively. Obesity is considered a modifiable risk factor for OA [39] and weight gain is a risk factor for undergoing TKA [40]. With the global increase of obesity [41], the number of overweight patients undergoing TKA is likely to increase. Obesity is associated with a variety of short and long term complications after TKA [42]. Consistent with our finding, in a systematic review [2], obesity was associated with a poorer quality of life after TKA. A recent study found that patients with a BMI ≥30 kg/m^2^ are at increased risk for poorer knee function, revision surgery within 5 years, superficial infection and deep venous thrombosis [43]. In addition, compared to non-obese patients, a systematic review found that patients with a BMI ≥40 kg/m^2^ had significantly lower implant survivorship and lower function scores. However, this association was not seen in patients with a BMI ≥30 kg/m^2^ [44]. Based on these findings, a cutoff BMI ≥40 kg/m^2^ may guide patient selection for TKA. However, Kulkarni and colleagues argued that because all patients are unique, the decision to perform surgery should not rely solely on a patient’s BMI but consider each patient’s potential risks and benefits [42]. Patients in this category may need counselling about weight reduction and other treatment alternatives for painful OA of the knee. Obese patients with BMI ≥30 kg/m^2^ needs to be informed about the risk of a poorer outcomes including SRH, prior to deciding to undergo TKA.

Patients with a higher number of painful sites before and 12 months after surgery were more likely to report poorer SRH, both prior to surgery and 12 months after TKA. This characteristic was the only factor common to both time points. Interestingly, number of comorbidities correlated with number of painful sites prior to surgery (.22, *p* = .008) but not with number of painful sites after 12 months (.10, *p* = .23). It is well known that patients with OA have a large number of painful sites often accompanied by pain sensitization [45] which can be linked to increased symptom severity. A higher number of painful sites as well as higher total symptom load was associated with persistent pain after TKA [46], lower functional scores [47], poor improvements in walking after TKA [21], poor overall health and sleep quality, female gender [48], and poorer health-related quality of life [49]. While preoperative widespread pain was associated with higher pain severity prior to and 12 months after joint replacement surgery [50], patients seem to gain the same amount of pain relief [50] and functional improvements [51] from their TKA compared to patients without widespread pain. Thus, multiple painful sites prior to surgery should be regarded as a risk factor for decreases in walking ability and poorer SRH 12 months after TKA.

Our study revealed that patients with higher preoperative depression scores were more likely to report poorer SRH at 12 months after TKA. This finding complements a systematic review of the literature stating that poorer mental health is associated with worse pain and functional outcomes 1 year after TKA [52]. However, in a recent study, depressed patients improved at the same or better rate as non-depressed patients and had similar satisfaction rates 1 year after TKA [53]. However, depressive symptoms are risk factors for poorer adherence with physical therapy [54]. Thus, identification of depressed patients may be important to individualize care plans and optimize their treatment outcomes.

Poorer SRH prior to surgery was significantly associated with poorer SRH 12 months after TKA. Similarly, poorer preoperative SRH was found to be predictive of poorer health outcomes and SRH 6 months following total hip and knee replacement surgery [55]. While in other samples, SRH was used as a predictor that captured a variety of future health outcomes [13–15], its use as a predictor in joint replacement surgery is limited. Because this simple tool is easy and non-demanding for clinical use, it could be considered for inclusion as part of a screening tool to assess patients at risk for poor outcomes after TKA surgery. However, more research is needed to establish its usefulness in conjunction with other risk factors.

Except for number of painful sites, the factors associated with SRH prior to surgery were not the same as the factors associated with SRH 12 months after TKA. Prior to surgery, SRH scores were associated with higher fatigue scores and higher levels of the inflammatory marker CRP. A systematic review found that higher CRP levels in OA patients were significantly associated with pain, decreased physical function, but not with radiographic OA, suggesting that low-grade systemic inflammation may increase patients’ symptomatology [56]. Patients with hip and knee OA are characterized by multiple co-occuring symptoms including pain, fatigue, and depression [57], which may impact their activity levels [58]. Hawker et al [59] found that fatigue contributed to lower physical activity and increased pain in OA patients, which in turn may lead to poorer SRH. However, as these findings were not factors in the model for SRH 12 months after TKA, they may be linked mainly to the OA condition and do not significantly impact the trajectory of recovery after surgery.

This study has several limitations. While our sample was recruited from one single hospital, the surgical department is a high volume surgical clinic which admits patients from all regions of Norway, which we believe increases the generalizability of our findings. No data were available on previous injuries that may have caused the OA condition. Other factors not accounted for in our study may contribute to long-term outcomes after TKA (e.g., inflammation, reactions to metal and polyethylene). More specific details on other chronic diseases 12 months after surgery may have provided us with more detailed information about other factors that impact patients’ SRH. Finally, the use of objective measures of walking ability may have provided additional insight.

The prospective design and relatively large sample are advantages with our study. In addition, all patients received the same type of implant and a standardized regimen for pain management and mobilization. Finally all of the patients received physical therapy for 4–6 months after TKA.

## Conclusions

In conclusion, patients with no improvements in terms of pain interference with walking report poorer SRH 12 months after TKA. In addition, poorer SRH after TKA was associated with higher BMI, a higher number of painful sites, higher depression scores and poorer preoperative SRH. Patients whose pain with walking over time does not improve need to be followed closely and may need additional physical therapy to improve their general health status. While a screening tool may enable clinicians to detect patients who need additional follow-up, more research is needed to develop and test such a screening tool. Finally, more research is needed to determine what type and dose of physical therapy is needed to improve TKA patients’ walking ability.

## Abbreviations

BIPQ: Brief Illness perception questionnaire
BMI: Body mass index
BPI: Brief pain inventory
CRP: C-reactive protein
FSS-7: Fatigue severity scale
GMM: Growth mixture modeling
HADS: Hospital anxiety and depression scale
LFS: Lee fatigue scale
NRS: Numeric rating scale
OA: Osteoarthritis
POD: Postoperative day
SRH: Self-rated health
TKA: Total knee arthroplasty
